# Identification of membrane-type 1 matrix metalloproteinase tyrosine phosphorylation in association with neuroblastoma progression

**DOI:** 10.1186/1471-2407-9-422

**Published:** 2009-12-04

**Authors:** Carine Nyalendo, Hervé Sartelet, Stéphane Barrette, Shigeru Ohta, Denis Gingras, Richard Béliveau

**Affiliations:** 1 Laboratoire de Médecine Moléculaire, Université du Québec à Montréal, C.P. 8888, Succ. Centreville, Montréal, Québec H3C 3P8, Canada; 2 Département de Pathologie, CHU Sainte-Justine, Montréal, Québec, Canada; 3 Département de Pédiatrie CHU Sainte-Justine, Montréal, Québec, Canada; 4 Department of Pediatrics, Shiga University of Medical Science, Otsu, Shiga, Japan

## Abstract

**Background:**

Neuroblastoma is a pediatric tumor of neural crest cells that is clinically characterized by its variable evolution, from spontaneous regression to malignancy. Despite many advances in neuroblastoma research, 60% of neuroblastoma, which are essentially metastatic cases, are associated with poor clinical outcome due to the lack of effectiveness of current therapeutic strategies. Membrane-type 1 matrix metalloproteinase (MT1-MMP, MMP-14), an enzyme involved in several steps in tumor progression, has previously been shown to be associated with poor clinical outcome for neuroblastoma. Based on our recent demonstration that MT1-MMP phosphorylation is involved in the growth of fibrosarcoma tumors, we examined the potential role of phosphorylated MT1-MMP in neuroblastoma progression.

**Methods:**

Tyrosine phosphorylated MT1-MMP was immunostained on tissue microarray samples from 55 patients with neuroblastoma detected by mass screening (known to be predominantly associated with favourable outcome), and from 234 patients with standard diagnosed neuroblastoma. In addition, the effects of a non phosphorylable version of MT1-MMP on neuroblastoma cell migration and proliferation were investigated within three-dimensional collagen matrices.

**Results:**

Although there is no correlation between the extent of tyrosine phosphorylation of MT1-MMP (pMT1-MMP) and MYCN amplification or clinical stage, we observed greater phosphorylation of pMT1-MMP in standard neuroblastoma, while it is less evident in neuroblastoma from mass screening samples (P = 0.0006) or in neuroblastoma samples from patients younger than one year (P = 0.0002). *In vitro *experiments showed that overexpression of a non-phosphorylable version of MT1-MMP reduced MT1-MMP-mediated neuroblastoma cell migration and proliferation within a three-dimensional type I collagen matrix, suggesting a role for the phosphorylated enzyme in the invasive properties of neuroblastoma cells.

**Conclusion:**

Overall, these results suggest that tyrosine phosphorylated MT1-MMP plays an important role in neuroblastoma progression and that its expression is preferentially observed in tumor specimens from neuroblastoma patients showing poor clinical outcome.

## Background

Neuroblastoma (NB) is the most common extracranial solid cancer in childhood, accounting for 15% of all cancer-related fatalities in children [1]. NB is a neuroendocrine malignant tumor of the autonomic nervous system that arises from neural crest cells [2]. NB shows heterogeneous pathological characteristics due to its variable sites of origin, diverse histopathologic appearance and biologic characteristics. This malignant tumor exhibits a broad spectrum of clinical features, including spontaneous regression without any treatment, maturation to a benign ganglioneuroma, or progression to metastasis leading to death. Numerous factors, such as patient's age at diagnostic, stage of disease, tumor histopathology and genetic abnormalities have all been shown to influence NB progression [3]. Among them, the metastatic status (Stage 4) is a very strong prognosis factor indicating poor outcome. Survival of children older than one year with an advanced stage of NB is poor despite aggressive treatment, as opposed to the survival of children younger than one year [4]. NB secretes catecholamine metabolites that are excreted in urine, offering a non-invasive diagnostic technique. This observation has led to the suggestion of screening for NB in infants using specific catecholamine markers. Large studies showed that mass screening did not appear to reduce neither the disease-related mortality nor the yearly numbers of aggressive NB [5-7]. Moreover, patients with mass screening-detected NB very rarely died from tumor progression [8]. Although the mechanisms underlying NB progression have been under intense investigation, therapeutic advances have failed to significantly increase the survival rates of children with aggressive NB.

Matrix metalloproteinases (MMPs), a family of zinc-dependent endopeptidases that degrade extracellular matrix (ECM) components, play crucial roles in several processes underlying tumor progression, including cell attachment, cell migration, invasiveness, cell proliferation, apoptosis, and angiogenesis [9-12]. There are 23 different human MMPs described to date, some of which are secreted in the pericellular environment while others are associated with the cell membrane. Membrane-type 1 matrix metalloproteinase (MT1-MMP, MMP-14), the best characterized membrane-anchored MMP, plays essential roles in tumor cell migration and invasion by acting as a potent matrix-degrading protease that digests a broad spectrum of ECM proteins [13-15] as well as a number of cell surface-associated adhesion receptors [16,17]. In particular, elegant studies have unequivocally shown that MT1-MMP-mediated pericellular proteolysis of the ECM is essential for tissue-invasive activity of tumor cells [18] as well as for tumor cell growth in an otherwise growth-restrictive three-dimensional (3-D) environment [19]. Accordingly, MT1-MMP is overexpressed in many types of tumors, including breast, colon, head and neck [20], cervical [21], gastric [22] and lung [23] carcinomas, as well as in brain tumors [24]. Moreover, MT1-MMP expression has been shown to correlate with unfavourable outcome in pediatric NB patients [25].

In addition to its important matrix-degrading activity, MT1-MMP contains a short cytoplasmic sequence that is involved in regulation of the enzyme activity [26] as well as in the activation of signal transduction processes [27,28]. Previous work from our laboratory has also shown that MT1-MMP is phosphorylated on its unique cytoplasmic tyrosine residue and that this phosphorylation also participates in tumor cell migration and invasion [29,30]. We have also shown that impaired tyrosine phosphorylation of MT1-MMP markedly reduced the tumorigenic properties of the highly invasive HT-1080 fibrosarcoma, leading to a complete inhibition of tumor growth in nude mice [31].

The aim of the present study is to investigate the role of tyrosine phosphorylated MT1-MMP (pMT1-MMP) in NB progression and its relation to clinical outcome. We report herein that tyrosine phosphorylation of the enzyme plays an important role in NB cell migration and proliferation within 3D collagen matrix *in vitro*. Furthermore, expression of pMT1-MMP correlated with some clinical features, patient age at diagnosis and mass screening versus standard NB, which are associated with poor prognosis in NB pediatric patients.

## Methods

### Patients

The study protocol have been performed with the approval of the CHU Sainte-Justine Ethic Committee of Research (protocol 2244), in accordance with the Helsinki Declaration. All tumor samples used in this study were obtained at diagnosis. Tumor samples from mass screening for NB were collected from 55 patients in two different countries: 35 came from Québec (Canada) and 20 from Otsu (Japan, [32]) (Table 1). Tumors were diagnosed between August 1989 and April 2002 with a median follow-up of 170 months. Among these 55 patients, there were 26 girls and 29 boys with a median age of 7 months. Three patients were older than one year at diagnosis and one tumor had MYCN amplification. According to the International Neuroblastoma Staging System classification [33], there were 16 patients in stage 1, 15 in stage 2, 9 in stage 3, 6 in stage 4 and 9 in stage 4S. One patient died from NB. Tumors samples from standard NB came from 234 patients with NB who were treated and followed in four centers: Bicêtre hospital (Le Kremlin-Bicêtre, France), Gustave Roussy Institute (Villejuif, France), the American Hospital (Reims, France) and CHU Sainte-Justine (Montréal, Canada). These patients were diagnosed between July 1988 and March 2008, with a median follow-up of 58.5 months. 167 patients were still alive at the last follow-up. There were 114 boys and 120 girls, with a median age of 25 months. Seventy three patients were under one year of age and 40 were older than 5 at the time of diagnosis (Table 1). There were 52 patients in stage 1, 32 in stage 2, 35 in stage 3, 97 in stage 4 and 18 in stage 4S. The global survival at the end of the follow-up was 91.7% in patients under one year, 65.2% in patient of one to five years and 52.5% in those of more than five years. Stage-related global survival (according to Kaplan-Meier method) was 94.2% in stage 1, 100% in stage 2, 85.7% in stage 3, 42.2% in stage 4 and 83.3% in stage 4S (Table 1).

**Table 1:** Clinical features of 289 patients with NB diagnosis

	Mass screening		Standard	
		
*Characteristics*	*Number (%)*	*Survival (%)*	*Numbers (%)*	*Survival (%)*
Total	55 (100)	98	234 (100)	71.3
Gender				
Female	26 (47.3)		114 (48.7)	
Male	29 (52.7)		120 (51.3)	
Age				
Median (range) in months	30 (0-151)		25 (0-183)	
< 1 year	52 (94.5)	98	73 (31.2)	91.7
1-5 years	3 (5.5)	100	121 (51.8)	65.2
> 5 years			40 (17)	52.5
Clinical stage				
1	16 (29.1)	100	52 (22.2)	94.2
2	15 (27.2)	93	32 (13.7)	100
3	9 (16.4)	100	35 (15)	85.7
4	6 (10.9)	100	97 (41.4)	42.2
4S	9 (16.4)	100	18 (7.7)	83.3
MYCN oncogene				
< 10 copies	54 (98.2)	100	160 (82.9)	79.6
> 10 copies	1 (1.8)	0	33 (18.1)	53.1

### Tissue Microarray (TMA) construction

On average, 4 tissue cylinders of 0.6 mm diameter were obtained and were transferred into a recipient paraffin block using a manual tissue arrayer (Alphelys, Plaisir, France). Mass screening NB samples consisted of 55 primary tumors and 21 metastases (15 lymph nodes and 6 hepatic metastases). TMA blocks of standard NB contained 234 primary tumors and 78 metastases (68 lymph nodes, 8 hepatic and 2 cutaneous metastases) and 56 paired control normal tissues (40 adrenal glands and 16 sympathetic ganglia).

### Immunohistochemistry

Five-μm sections of TMA blocks were cut and deparaffinized, treated with 1% H_2_O_2 _and submitted to antigen retrieval by microwave oven treatment for 15 min in citrate buffer. Immunohistochemical staining was performed on these sections using pMT1-MMP polyclonal antibodies (1/100, 1 hour at room temperature [29,31]). Samples were then incubated with biotinylated immunoglobulin (LSAB II, DAKO, Glostrup, Denmark) at room temperature for 30 min followed by avidin-biotin peroxidase complexes for 30 min. Rabbit IgG was used as a negative control. Three-amino-9-ethylcarbazol was used as the chromogen and haematoxylin as the nuclear counterstain. TMA sections were treated in one set of immunostaining. Two pathologists, blinded from clinical data, independently evaluated immunostaining under a light microscope at a magnification of 400×. Immunostaining scores were established by a semi-quantitative optical analysis of samples containing more than 10 neuroblasts assessing the percentage of positive cells in each sample: 0 = all cells negative, 1+ = 1 to 25% of positive cells, 2+ = 26 to 50% of positive cells, 3+ = 51 to 75% of positive cells and 4+ more than 76% of positive cells. Inter-observer agreement was calculated by the kappa coefficient. The observed agreement between the two pathologists was Kappa = 0.61, 95% CI = [0.58-0.64], P < 0.0001.

### Statistical analysis

The univariate relationship between immunohistochemical expression of pMT1-MMP and biological and clinical data (standard and mass screening NB, control samples, primary tumors, metastasis, stage 1 and stage 4 and patient under one year, MYCN amplification) was investigated by Student's t-test. Kaplan-Meier analysis was used to estimate cancer-specific survival and the groups were compared with the log-rank test. All analyses were performed with Graphpad Prism software. P < 0.05 was considered statistically significant.

### Cell lines and transfection

NB-10 cells were from St Jude Children's Research Hospital (Memphis, TN, USA) and were a gift from Dr. Daniel Sinnett (CHU Sainte-Justine, Montreal). SK-N-SH cells were obtained from the American Type Culture Collection. Cells were cultured at 37°C in a humidified atmosphere composed of 95% air and 5% CO_2 _and were grown in Eagle's minimum essential medium (EMEM) supplemented with 1 mM sodium pyruvate and 10% fetal bovine serum (FBS). The cDNAs encoding the full-length human MT1-MMP (MT1 WT) and its cytoplasmic mutant (MT1 Y573F) have been previously described [29]. NB-10 and SK-N-SH neuroblastoma cells were transiently transfected using the FuGENE HD transfection reagent (Roche Applied Science) according to the manufacturer's instructions.

### Western Blotting

The procedures used were described previously [34]. Briefly, equal amounts of protein from cell lysates were solubilized in Laemmli sample buffer, boiled for 5 min, separated by SDS-PAGE, transferred onto PVDF membranes and immunodetected with antibodies against MT1-MMP (MAB3328, Chemicon International) or GAPDH (Santa Cruz Biothechnology, CA, USA). For phosphorylated MT1-MMP assessment, cell lysates were immunoprecipitated with MT1-MMP antibodies (MAB3328) prior to Western blot procedure and immunodetection with pMT1-MMP polyclonal antibodies [29].

### Cell Migration Assay

Migration assays were performed on transwells pre-coated with 10 μg/ml type I collagen. Transwells were assembled into 24-well plates and the lower chambers were filled with EMEM containing 10% FBS. Transfected cells were harvested, resuspended in 100 μl of fresh serum-free EMEM at a density of 5 × 10^5 ^cells/ml, and inoculated into the upper chamber of each transwell. The plates were then placed at 37°C in 5% CO2, 95% air for 3 h, and cells that had migrated were quantified using computer-assisted imaging (Northern Eclipse 6.0; Empix Imaging, Mississauga, ON, Canada). Statistical analyses were performed by one-way analysis of variance (ANOVA) followed by Bonferroni post-tests using GraphPad Prism software. P < 0.05 was considered statistically significant.

### 3D cell proliferation

Type I collagen was extracted from rat tail and resuspended at 2.7 mg/ml in acetic acid. Cells were mixed with 10× EMEM, 0.17N NaOH and 2.7 mg/ml type I collagen. The mixture was allowed to gel for 45 min at 37°C and EMEM containing 10% FBS was added atop. Collagen gels were dissolved with 2 mg/ml bacterial collagenase (Sigma, Burlington, ON, Canada) and viable cells were counted by trypan blue exclusion using a hemacytometer. Statistical analyses were performed by one-way ANOVA followed by Bonferroni post-tests using GraphPad Prism software. P < 0.05 was considered statistically significant.

### Fluorescence and Confocal Microscopy

NB-10 cells were seeded on 10 μg/ml type I collagen-coated cover-slips, serum-starved overnight and stimulated with serum for 15 min. Cells were then fixed with 3.7% formaldehyde for 15 min, incubated for 5 min with 20 μM Hoechst (Sigma) for nuclei staining, permeabilized with 0.2% Triton X-100 for 5 min and blocked (1% BSA in Tris Buffered Saline containing 0.1% Tween 20) for 30 min. Samples were then incubated with FITC-conjugated phalloidin (for actin staining) for 30 min, and stained with specific primary antibodies against pMT1-MMP [29] for 30 min, followed by a 30 minute incubation with rhodamine-conjugated secondary antibodies (Molecular Probes - Invitrogen, Burlington, ON, Canada). Samples were covered with Immuno-Fluor Mounting Medium (MP Biomedicals, Solon, OH, USA). Fluorescence was visualized and photographed using a Zeiss LSM 510 Meta confocal microscope.

## Results and Discussion

### Phosphorylation of MT1-MMP in NB specimens

NB is characterized by its variable evolution that allows separation of these tumors into two major groups. Tumors in the first group are relatively benign, localized, well-differentiated and are successfully treated by surgery alone, or they sometimes regress spontaneously. Tumors in the second group (60% of all NB) are invasive and metastatic, and are usually associated with poor clinical outcome in spite of intensive treatment [35]. Tumors detected by mass screening are usually part of the first group and are associated with good clinical outcome. Notwithstanding many advances in the identification of the molecular factors associated with the poor clinical income of NB, including amplified expression of the *MYCN *oncogene, deletion of 1p or 11q [36], lack of CD44 expression [37] as well as expression of neurotrophin receptors [2], the molecular mechanisms responsible for the progression/regression evolution of NB are poorly understood.

**Figure 1:**
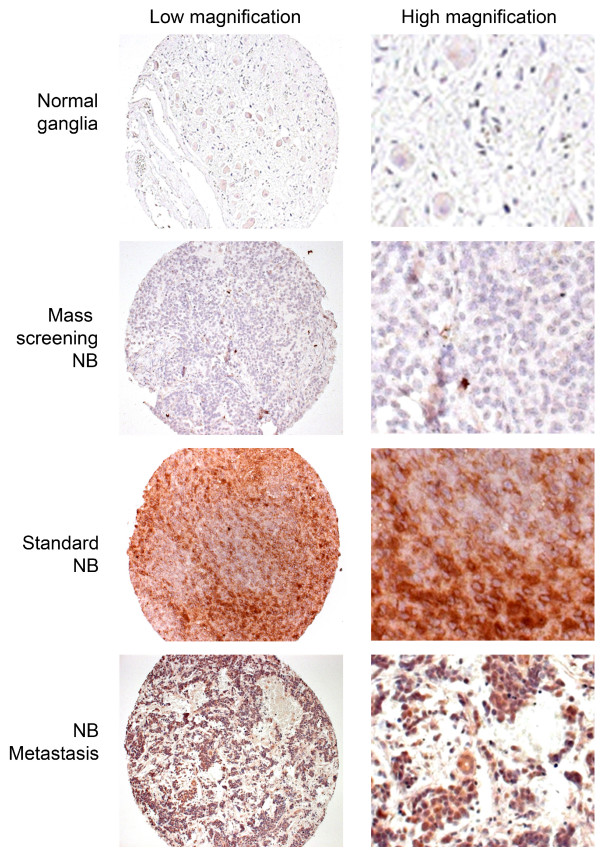
**Immunostaining of pMT1-MMP in NB samples**. pMT1-MMP was immunostained in a tissue microarray of NB specimens.

Since a previous study showed correlation between MT1-MMP expression and advanced stage of NB and patients survival [25], we examined in this study whether phosphorylation of MT1-MMP was involved in NB progression. 289 NB samples (Table 1) were immunostained with pMT1-MMP antibodies [29,31]. The phosphorylated MT1-MMP was membranous and cytoplasmic and was only found in neuroblastic cells (Figure 1). As shown in Figure 1, normal control samples presented a poor and infrequent phosphorylation of MT1-MMP (33.9% of sample, means intensity 0.34), NB had a moderate and frequent phosphorylation of MT1-MMP (64.7% of sample, means intensity 0.72) similar to those of metastases (73.8% of sample, means intensity 0.72)

### Relationship between pMT1-MMP and clinicopathologic parameters

We next evaluated the correlation between pMT1-MMP immunostaining and clinicopathologic features (Table 2). Analysis of immunostaining showed that MT1-MMP exhibits greater phosphorylation in NB specimens compared to control samples (P < 0.0001). More precisely, immunostaining of pMT1-MMP was observed in 187 of 289 (64.7%) of NB specimens, while only 19 of 56 (33.9%) of normal specimens were positive for MT1-MMP. pMT1-MMP detection was significantly lower in mass screening NB (24 of 55 (43.6%)) than in standard NB (160 of 234 (68.4%), P = 0.0006). Moreover, pMT1-MMP detection was higher in patients older than 1 year than in those under one year (P = 0.0002). However, no significant relationship was observed between pMT1-MMP and the tumor site (primary versus metastasis, P = 0.5900), clinical stage (stage 1 versus stage 4, P = 0.3700) or MYCN amplification (amplified versus no amplified P = 0.1000).

**Table 2:** Univariate analysis of pMT1-MMP expression in clinicopathologic features

*Characteristics*	N*	*Mean intensity*	*Positive* *N (%)*	*Negative* *N (%)*	*P*
Type of samples					
Normal control samples	56	0.34	19 (33.9)	37 (66.1)	
Tumor from primary site	289	0.72	187 (64.7)	102 (35.3)	0.0001
Tumor from metastasis	103	0.72	76 (73.8)	27 (26.2)	0.5900
Age					
< 1 year	125	0.64	58 (46.4)	67 (53.6)	0.0002
> 1 year	184	0.78	124 (67.4)	60 (32.6)	
Clinical stage					
1	68	0.66	41 (60.3)	27 (39.7)	0.3700
4	103	0.75	69 (67.0)	34 (33.0)	
Specimen type					
Mass screening	55	0.31	24 (43.6)	31 (56.4)	0.0006
Standard	234	0.8	160 (68.4)	74 (31.6)	
MYCN oncogene					
Amplified	34	0.52	21 (61.8)	13 (38.2)	0.1000
Not amplified	214	0.69	100 (46.7)	114 (53.3)	

*N: number of specimens

### A non phosphorylable mutant of MT1-MMP inhibits MT1-MMP-mediated tumorigenic properties of NB *in vitro*

**Figure 2:**
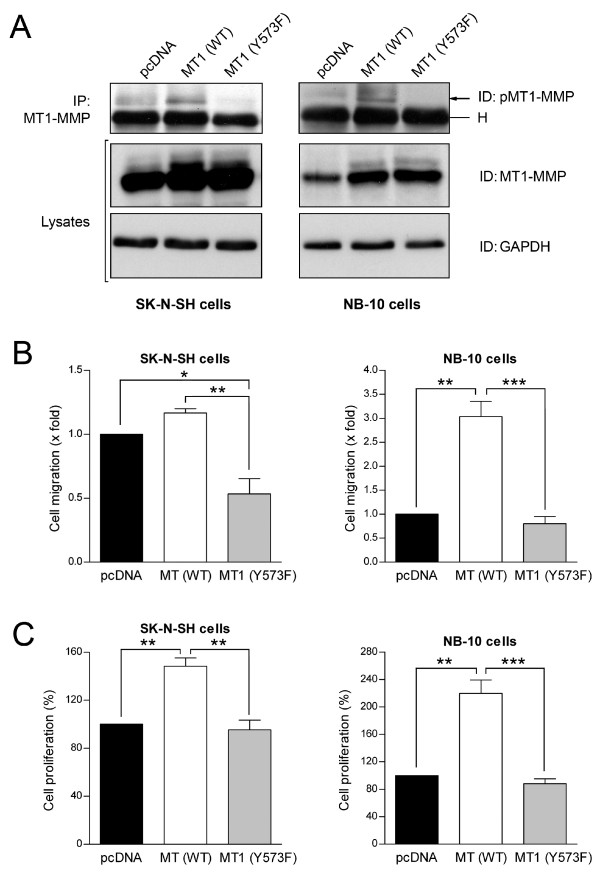
**Inhibition of neuroblastoma cell proliferation and invasion by a non-phosphorylable mutant of MT1-MMP**. NB-10 and SK-N-SH neuroblastoma cells were transfected with either pcDNA, WT or Y573F forms of MT1-MMP. 24 h post-transfection, cells were harvested and transfection efficiency as well as MT1-MMP phosphorylation levels were determined by Western blot (A); IP: immunoprecipitation, ID: immunodection, H: immunoglobulin heavy chain. (B) 5 × 10^4 ^transfected cells were subjected to migration assays, using Transwells pre-coated with 10 μg/ml type I collagen and 10% serum as chemoattractant. (C) 2 × 10^4 ^transfected cells were embedded within type I collagen gels and allowed to grow during 5 days. Data are means ± SEM. *P < 0.05; **P < 0.01; ***P < 0.001.

We have previously shown that tyrosine phosphorylation of MT1-MMP, in addition to affecting its proteolytic activity, plays a critical role in the tumorigenicity of fibrosarcoma cells [29,31]. To determine the role of phosphorylated MT1-MMP in NB *in vitro*, NB-10 and SK-N-SH NB cells were transiently transfected with either wild type (WT) or the non phosphorylable version (Y573F) of MT1-MMP. As shown in Figure 2A, due to elevated basal expression of MT1-MMP in SK-N-SH cells, overexpression of WT MT1-MMP in these cells had a relatively low stimulatory effect on cell migration while the Y573F mutant strongly diminished both cell migration and MT1-MMP phosphorylation (Figure 2A-B). However, in the NB-10 cells which express low basal levels of MT1-MMP, overexpression of WT MT1-MMP markedly increased cell migration (3-fold), this effect being completely abolished by overexpression of the Y573F mutant (Figure 2B). These results are in agreement with previous findings showing the dominant-negative properties of the non phosphorylable mutant of MT1-MMP [29,31].

**Figure 3:**
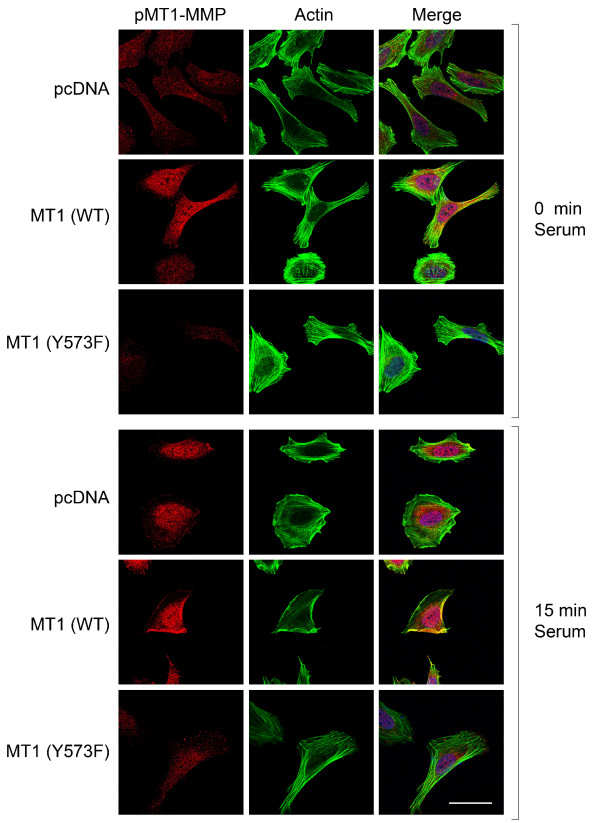
**Localization of tyrosine phosphorylated MT1-MMP in NB cells**. NB-10 neuroblastoma cells were transfected with either pcDNA, WT or Y573F forms of MT1-MMP. 24 h post-transfection, cells were seeded on 10 μg/ml type I collagen-coated cover slips, serum-starved for 18 h, stimulated for 15 min with serum and fixed with formaldehyde. Nuclei, actin and pMT1-MMP were stained as described in Material and Methods. Images were acquired using a Zeiss LSM 510 Meta confocal microscope. The white scale bar represents 50 μm.

We next evaluated the impact of MT1-MMP phosphorylation on NB proliferation within 3D collagen matrices. As shown in Figure 2C, overexpression of the wild type version of the enzyme induced a marked increase in the proliferation of both SK-N-SH and NB-10 cells within 3D collagen matrices, this stimulatory effect being abolished by the non phosphorylable MT1-MMP mutant. Previous study from our laboratory demonstrated that the inhibitory effect of the Y573F dominant-negative mutant of MT1-MMP on tumor cell proliferation is not related to defective proteolysis, since this mutant retained the ability to activate proMMP-2 and to degrade type I collagen [31]. The inhibitory effect of the Y573F mutant on NB cell migration and proliferation correlated with a decrease in the levels of phosphorylated MT1-MMP (Figure 3). Moreover, serum-induced relocalization of tyrosine phosphorylated MT1-MMP to peripheral actin-rich structures, a process important for cell migration, was completely abolished by overexpression of the non-phosphorylable mutant of MT1-MMP (Figure 3, [30]).

These results suggest that phosphorylated MT1-MMP may play an important role in the MT1-MMP-mediated pro-migratory and pro-invasive properties of NB *in vitro*.

## Conclusion

Overall, the findings reported in this study show that reduction of the tyrosine phosphorylation of MT1-MMP reduced the MT1-MMP-mediated tumorigenic properties of NB cells *in vitro*, and that pMT1-MMP is preferentially expressed in tumor specimens from standard NB and in patients older than one year, two clinical features associated with poor outcome in NB patients. In addition, our previous findings suggest that phosphorylation of MT1-MMP is observed under tumorigenic rather than normal conditions [31]. Therefore, phosphorylation of MT1-MMP could represent the ''molecular switch" in NB cells responsible for the evolution from benign to malignant tumor. The identification of pMT1-MMP as an important player in the process of NB progression could be useful for the development of new therapeutic strategies for NB patients who cannot be cured with current therapeutic approaches.

## Competing interests

The authors declare that they have no competing interests.

## Authors' contributions

CN participated in the design of the study, carried out the *in vitro *experiments, participated in tissue microarray analysis and drafted the manuscript. HS carried out and analyzed the tissue microarray staining and performed the statistical analysis. SB and OS participated in the tissue microarray construction. DG participated in the design and coordination of the manuscript and helped to draft the manuscript. RB conceived of the study, and participated in its design and coordination. All authors read and approved the final manuscript.

## Pre-publication history

The pre-publication history for this paper can be accessed here:

http://www.biomedcentral.com/1471-2407/9/422/prepub
